# Development and Validation of a Virtual Version of the Box and Block Test to Assess Manual Dexterity at Home for Adults with Stroke and Children with Cerebral Palsy

**DOI:** 10.3390/bioengineering12060662

**Published:** 2025-06-16

**Authors:** Zélie Rosselli, Merlin Somville, Edouard Ducoffre, Carlyne Arnould, Geoffroy Saussez, Yannick Bleyenheuft

**Affiliations:** 1 MSL-IN Laboratory, Institute of neuroscience, UCLouvain, 1200 Bruxelles, Belgium; zelie.rosselli@uclouvain.be (Z.R.); merlin.somville@uclouvain.be (M.S.); saussezg@helha.be (G.S.); 2 Forme & fonctionnement Humain (FfH) Laboratory, CeREF-Santé, Haute Ecole Louvain en Hainaut, 6061 Charleroi, Belgium; ducoffree@helha.be (E.D.); arnouldc@helha.be (C.A.)

**Keywords:** patient outcome assessment, dexterity, hand function, virtual device, telerehabilitation, neurological disorder, stroke, cerebral palsy, motor skills, executive function

## Abstract

The REAtouch^®^ Lite device was recently developed to support motor skill learning-based interventions, integrating both games/activities and assessment tools to enable home-based telerehabilitation. Given the importance of hand functions in rehabilitation of patients with brain lesions, this study aimed to validate a virtual version of the Box and Block Test (vBBT) implemented in the REAtouch^®^ device. A total of 205 healthy participants, 37 post-stroke adults, and 37 children with cerebral palsy (CP) performed the standard BBT, various versions of the newly designed vBBT (with/without a separation wall; with 6, 4, and free zones) and the Tower of London test assessing executive function/planning abilities. Friedman’s ANOVA revealed significant differences between the BBT and all versions of the vBBT scores in healthy participants (all *p* < 0.001). However, the vBBT-4 zones showed the largest intraclass correlation coefficient (ICC) with the BBT in healthy participants (0.58) and even higher correlations in participants with CP and stroke (>0.8). Only the vBBT-6 zones version showed a significant correlation with patients’ planning abilities (*p* < 0.01; r = −0.28). These findings highlight the vBBT-4 zones as the most relevant version to assess hand dexterity directly with the REAtouch^®^ device, potentially within telerehabilitation modalities. Further normative data must be established.

## 1. Introduction

Neurological disorders are increasingly acknowledged as a significant global cause of mortality and disability, affecting both adults and children [1]. Cerebral palsy (CP) and stroke represent the primary causes of physical disability, respectively, in the pediatric and adult populations [2,3]. CP is a non-progressive brain lesion that typically occurs in early childhood, before the age of two, and is characterized by motor impairments (and associated non-motor symptoms, i.e., pain or intellectual disabilities) [2]. Similarly, stroke is characterized by an abrupt brain dysfunction occurring in a mature brain, due to vascular events, with outcomes ranging from full recovery to severe, chronic disability. The most common post-stroke complications include hemiparesis, sensory deficits, visuospatial neglect, and spasticity [4]. All of these have a significant impact on independence and quality of life [4]. A common challenge faced by both CP and stroke patients is the impairment of manual dexterity [5,6,7]. Dexterity is defined as the ability to perform coordinated, fine hand and finger movements that are essential for manipulating objects through the integration of muscular, skeletal, and neurological systems [6,7,8]. Consequently, dexterity impairments have a large impact on an individual’s ability to perform activities of daily living independently [9]. It is estimated that over half of the stroke survivors continue to experience motor deficits, including reduced dexterity, despite undergoing conventional rehabilitation [10]. Similarly, approximately 60% of children with CP aged 4 to 14 experience significant hand dysfunctions [11]. It is therefore essential to address these deficits to potentially enhance patients’ independence. Some therapeutic interventions have demonstrated efficacy to improve manual dexterity and its transfer in daily life to increase functional performance, including constraint-induced movement therapy (CIMT), hand–arm bimanual intensive therapy (HABIT), and hand–arm bimanual intensive therapy including lower extremities (HABIT-ILE) [12,13]. However, these approaches are frequently provided in clinical settings, which can impede accessibility for numerous patients due to logistical and financial constraints [14,15]. To address these challenges, at-home versions of such evidence-based interventions have emerged as innovative solutions [16,17]. These remote rehabilitation approaches have the potential to reduce travel requirements and offer greater flexibility, enabling patients to participate in therapy from their homes. This study will focus on an ongoing project designed to implement a 2-week HABIT-ILE intervention in a home-based telerehabilitation setting in children with CP and adults with stroke [18,19]. In order to facilitate remote therapy and supervision, the REAtouch^®^ Lite device has been developed as an interactive, portable device that enables patients to engage in rehabilitative activities under the remote supervision of trained therapists. In addition to offering a home-based rehabilitation opportunity, our aim was also to develop remote assessment [20,21]. Motor learning, a central aspect of therapies such as CIMT, HABIT, and HABIT-ILE, is typically evaluated through the administration of standardized tests. These tests serve to guide treatment plans, monitor progress, and assess the effectiveness of interventions. Among these, the Box and Block Test (BBT) is a widely used tool for evaluating gross manual dexterity, given its ease of use, correlations with daily life activities requiring upper extremity abilities, and good psychometric qualities with excellent reliability and test–retest validity [22,23].

The aim of this study is to develop and validate a virtual version of the BBT directly using the REAtouch^®^ device, namely, the virtual Box and Block Test (vBBT). The validation of vBBT versions will be investigated by correlations with the original BBT. In addition, the potential influence of participants’ executive functions and planning abilities on the scores will be taken into account.

## 2. Materials and Methods

### 2.1. Ethics

This multicentric concurrent validity study received approval from the Ethics Committee of the Saint-Luc-UCLouvain Hospital Faculty in Belgium and is registered on ClinicalTrials.gov (B4032022000142).

### 2.2. Sample Size

Sample size estimations were performed using PASS 14 software (NCSS, LLC. Kaysville, UT, USA). The sample size calculation of healthy participants was based on achieving a two-sided 95% confidence interval with a width of 0.299, assuming an intraclass correlation coefficient of 0.500 between the conventional and virtual versions of the BBT. An overall sample of 98 participants was required.

The sample size calculation of participants with CP or stroke was based on achieving a two-sided 95% confidence interval with a width of 0.296, assuming an intraclass correlation coefficient of 0.800. An overall sample of 27 participants was required.

### 2.3. Participants

All participants were informed and signed a consent form (for adults) or an assent form (for children), along with a consent from their legal tutor(s).

Healthy participants in phases 1 and 2 were recruited through social media platforms and information sessions held in the auditoriums of UCLouvain and HELHa. The inclusion and exclusion criteria were the same for both phases. The inclusion criteria were individuals aged 18 and over, while exclusion criteria were the presence of upper limb deficits, a history of neurological disorders, and uncorrected visual impairments. All participants performed the tasks using only their dominant hand, following the same standardized protocol.

In phase 3, participants with CP or stroke were recruited to participate in larger randomized controlled trials from the BioWinproject “FRITE@Home” [18,19]. To be included, participants with CP had to be aged between 5 and 18 years and demonstrate sufficient cognitive ability to follow simple instructions (e.g., throwing a ball). Individuals with uncontrolled seizures, botulinum toxin injections, intensive therapy, orthopedic surgery within the three months preceding the assessment, or severe visual or cognitive impairments were excluded.

For participants with a stroke, the inclusion criteria were being aged 18 or older, with a history of chronic stroke (≥6 months post-stroke), able to follow simple instructions, and able to initiate shoulder movement. Individuals who met the following exclusion criteria were not eligible to participate in the study: recent botulinum toxin injections, intensive therapy, or surgery within the past three months, or uncontrolled seizures.

### 2.4. Data Collection Protocol

This study was performed in three subsequent phases. Each study phase involved an independent participant sample. The study was not designed as a longitudinal follow-up. The first two phases were conducted using healthy subjects, whereas the third phase involved individuals with cerebral lesions. The order of administration of the different test versions was randomized across participants to minimize potential order and learning effects.

The first phase aimed to determine the best hardware setting for the new vBBT developed on the REAtouch^®^ by testing the impact of using or not using a separating wall between the two compartments, along with the standardized setup of the BBT. This phase was implemented by testing only the dominant hand.

Based on the results observed in the first phase, the second phase was performed to determine the best version of the vBBT based on the software itself and the instructions given to the participants. Three different versions of the vBBT were tested (6 zones, 4 zones and a free zone), along with the realization of an assessment for motor planning (i.e., the Tower of London test). Once again, only the dominant hand was tested during this phase.

For the third phase of this study, individuals with a stroke or bilateral CP completed the chosen version of the vBBT and the BBT with both hands. To further test the concurrent validity of the vBBT, participants also completed an ABILHAND questionnaire assessing the performance in daily life activities requiring the use of hand functions for adults with stroke (ABILHAND-CS) and children with CP (ABILHAND-Kids).

### 2.5. Materials

**Figure 1:**
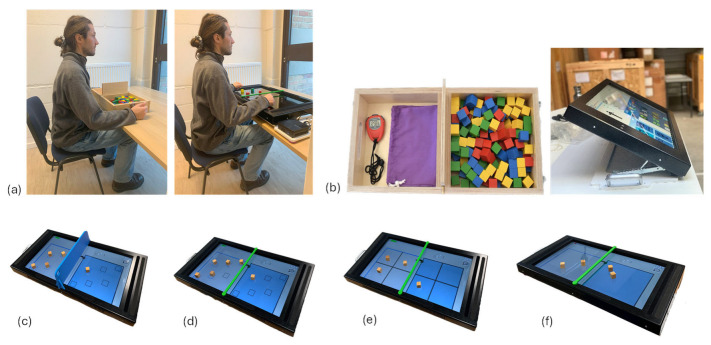
**Material (a) Standardized test position**. Participant is seated with hips, knees, and ankles bent at 90 degrees, feet flat on the floor, table positioned at elbow height. (**b**) **Original Box and Block Test (BBT) and REAtouch^®^ Lite device**. REAtouch^®^ Lite is a virtual rehabilitation tool designed to support upper limb training through interactive games and activities. Interaction with the touchscreen is achieved using tangible objects. **Different versions of the vBBT**. One version comprises (**c**) six zones and a high plastic separation, while the remaining three versions include a low bar separation with (**d**) six zones, (**e**) four zones, and (**f**) a free zone.

All of the BBT tests began with a preliminary trial prior to the start of the 60 s test. All tests were conducted in a standardized position, with participants seated with their hips, knees, and ankles flexed at 90 degrees, feet flat on the floor, table set at elbow height (Figure 1a). For individuals with a history of stroke or bilateral CP who had not yet achieved independent control of their trunk, the assessments were conducted with the use of a back support in a chair with a backrest or their own wheelchair.

Box and Block Test

The BBT consists of a rectangular box divided by a central partition into two identical compartments, measuring 25.5 × 25.5 cm [22]. The setup contains standardized 150 blocks, measuring 2.5 × 2.5 cm (Figure 1b). During the test, participants are instructed to transfer as many blocks as possible from one compartment to the other within 60 s, using only one hand. Each block must be transferred individually, crossing the center line of the box with the fingertips before being released. If several blocks are moved by mistake, only one is counted. When both hands are tested, the less-affected hand (LAH) is tested first, followed by the same protocol for the more-affected hand (MAH). A 15 s training trial is performed before the start of the test. The final score is the total number of blocks transferred between compartments within the 60 s time limit. The BBT was used in the three phases of this study.

Virtual Versions of the Box and Block Test (Table 1)

All of the vBBT versions are performed using the REAtouch^®^ Lite device. The REAtouch^®^ is a medical device developed by Axinesis S.A. (Wavre, Belgium). The device consists of an interactive interface including a large touch screen, allowing interaction with tangible object manipulation. Equipped with the TouchLAB software (Version 2.8.0), REAtouch^®^ offers a wide range of games/activities and a developing catalog of assessment tools. The REAtouch^®^ Lite is a portable version of the device designed for remote use, enabling rehabilitation sessions to be conducted at home. It has a 32-inch screen (73 × 41 cm) and weighs 15 kg. The full device measures 100 cm long, 55 cm wide, and 8 cm high, with an adaptable tilt of the screen from 0° to 80° (Figure 1b).

**Table 1:** Summary of the characteristics of the three vBBT versions.

Version	Number of Zones	Type of Separator	Number of Blocks Used	Practice Trial	Error Rate (%)
vBBT—6 zones	6 (4 × 4 cm)	High wall (13.5 cm) or low bar (2 cm)	6	24 blocks	Not reported
vBBT—4 zones	4 (14 × 14 cm)	Low bar only (2 cm)	4	16 blocks	1.32
vBBT—Free zone	*None*	Low bar only (2 cm)	4	16 blocks	7.84

During the vBBT test, the screen is maintained in a flat position and divided into two 29 × 29 cm areas. For every version of the vBBT, participants have to transport as many blocks as possible from one side to the other and back again; that is, they perform as many back-and-forth movements as possible in 60 s, using only one hand. Blocks are the same as those used for the Box and Block Test (2.5 × 2.5 cm), and the final score is the total number of blocks transferred within the 60 s time limit.

#### 2.5.1. Study Phase 1

For this phase, a vBBT-6 zones version was developed on the REAtouch^®^ and performed with either a separation wall imitating the central wall of the BBT (13.5 cm height, 0.8 cm width) or a low separating bar (2 cm height, 1.5 cm width) (Figure 1c,d). For this version of the test, each of the two large areas included six smaller zones (4 × 4 cm). At the start of the test, six blocks are placed onto the six zones in front of the hand being tested. Participants are instructed to move the blocks back and forth from one large area to the other, placing them in the 6 small zones in order to complete as many transfers as possible within 60 s. The last (6th) block moved from one area to the other must be fully released before the next block transfer can begin. There is no specific order for moving the blocks. Participants benefited from a trial period with 24 blocks to practice.

#### 2.5.2. Study Phase 2

The second part of the study was completed using solely the bar between the two large areas of the screen. In addition to the 6 zones version, 4 zones and free zone versions were tested (Figure 1d–f). The vBBT-4 zones version was performed with 4 blocks, with the two large areas of the screen split into 4 smaller zones of 14 × 14 cm. As in the previous version, participants had to transport as many blocks as possible from one side of the screen to the other within 60 s. The last (4th) block moved from one area to the other must be fully released before the next block transfer can begin. Participants had a trial period with 16 blocks to practice.

The vBBT-free zone is identical to the previous one, except that the zones have been removed, leaving only the two large areas, where blocks can be placed freely rather than in designated zones.

For all virtual versions, in healthy participants, in addition to the score displayed by the REAtouch^®^ Lite device, the number of blocks transported was also manually counted by the examiner using a clicker in order to measure the error rate (in %) of the REAtouch^®^ Lite in detecting the blocks. This was calculated using the following formula:

(1) $$\left(\frac{\mathrm{Assumed}\;\mathrm{value}-\mathrm{Actual}\;\mathrm{Value}\;}{\mathrm{Actual}\;\mathrm{value}}\right)\times 100$$

The assumed value corresponds to the score displayed by the REAtouch^®^ Lite, whereas the actual value is the one recorded by the examiner. These error rates were used to evaluate the system’s technical accuracy.

Tower of London

The Tower of London (ToL) test is a neuropsychological test designed to assess executive functions, particularly planning ability [24]. It consists of a board with three pegs and three colored beads, along with 14 cards. Twelve out of the fourteen cards are used for the assessment, while the remaining two represent the starting position and a practice attempt. Each card shows a specific alignment of colored balls. The test consists of reproducing this alignment with the pegs and colored beads in a given maximal number of moves. Two measures are recorded: the time needed to plan the first move (planning time) and the time needed from the first move to the last move required to resolve the model (execution time). Total scores were determined by summing the recorded planning times for all twelve models.

#### 2.5.3. Study Phase 3

For the third part of the study, the BBT and the vBBT-4 zones were tested in children with bilateral CP and adults with stroke. Both hands were tested, starting with the LAH.

ABILHAND Questionnaires

Manual performance, defined as the capacity to accomplish daily tasks involving the upper limbs, regardless of the strategies employed and the limbs used, was evaluated using an ABILHAND questionnaire. ABILHAND-Kids was used in children with CP as it was specifically adapted and validated for this population. Caregivers, primarily parents, assessed their child’s ability to complete 21 manual activities, indicating the level of ease or difficulty encountered. This questionnaire, based on the Rasch model, provided a linear measurement expressed in logits, ranging from 0% (minimal manual performance) to 100% (maximal manual performance). ABILHAND-Kids demonstrated strong validity, reliability, and sensitivity to change in children with CP [25,26]. For the adult stroke population, manual performance was assessed using ABILHAND-CS, a version specifically adapted and validated for individuals with chronic stroke. Unlike the pediatric version, ABILHAND-CS was completed directly by the patients themselves, allowing for a self-reported evaluation of their perceived ease or difficulty in performing 23 bimanual activities [27].

### 2.6. Statistical Analysis

Statistical analyses were performed using the SPSS software (Version 29.0.2.0 (20)), with a significance level of 0.05 for all tests.

#### 2.6.1. Study Phase 1

Friedman’s ANOVA was used to assess differences between the two 6-zones versions of the vBBT and the original BBT in healthy participants, as the assumption of homogeneity of variances was not met. A post hoc test was then used to compare each group individually.

To assess the validity of each version versus the original BBT scores, intraclass correlation coefficients (ICCs) were calculated using a two-way mixed model with homogeneity type. Correlation strength was classified as low (0.1 ≤ r < 0.3), moderate (0.3 ≤ r < 0.5), or large (r > 0.5), following Cohen’s conventions [28].

#### 2.6.2. Study Phase 2

The same statistical approach (Friedman’s ANOVA and ICCs) was applied to assess differences and validity between the three vBBT versions with low bar separation and the original BBT in healthy participants.

Additionally, Spearman correlations were used to examine the correlation between ToL planning scores and the various BBT and vBBT scores, as the normality assumption for ToL scores was not met (*p* < 0.001, Kolmogorov–Smirnov test).

#### 2.6.3. Study Phase 3

For individuals with stroke and bilateral CP, ICCs were calculated between the vBBT-4 zone and the original BBT for both LAH and MAH using the same two-way mixed model with homogeneity.

Depending on data normality, Spearman’s or Pearson’s correlation was used to examine the relationship between ABILHAND and vBBT scores.

## 3. Results

### 3.1. Participants

Demographic data for all participants are displayed in Table 2. A hundred healthy individuals participated in the first phase of the study. A group of 105 other healthy participants took part in the second phase of the study. Finally, 37 children with bilateral CP (mean age 9.6 years, MACS range: I–IV, GMFCS range: I–V) and 37 adults with chronic stroke (mean age 61.7 years, mRS range 1–5, MoCA range: 12–30) participated in the third and last phase of the study.

### 3.2. Study Phase 1

**Figure 2:**
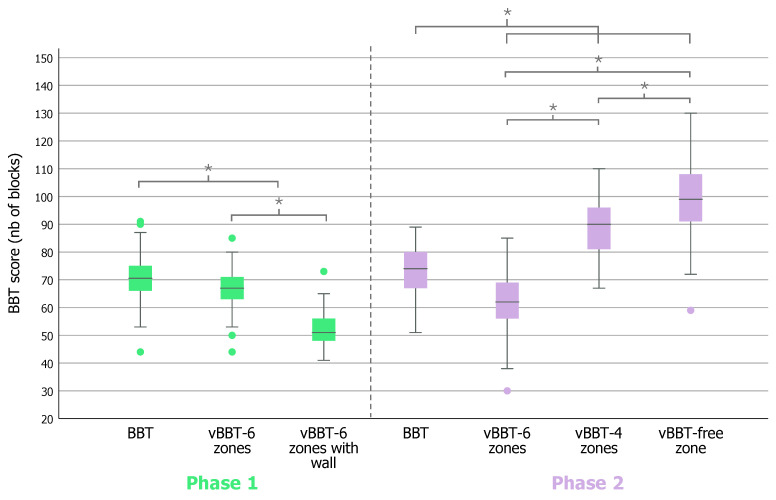
**BBT and vBBT scores, phases 1 and 2.** Box plots showing the distributions of the scores (number of blocks per minute) observed in the different versions of the Box and Block Test (BBT). The line inside the box represents the median, the box shows the interquartile range, the whiskers are the minimum and maximum values that are not outliers, and the dots indicate extreme values (>1.5 times the interquartile range). *vBBT = virtual Box and Block Test, nb= number*; * = *p* < 0.001.

During phase 1, we observed a significant difference between the BBT and the two 6-zone versions, with and without the separation wall (*p* < 0.001) (Table 3, Figure 2). A higher number of blocks were transported in 60 s during the original test compared to the vBBT versions. Moreover, as shown in Figure 2, the vBBT-6 zones with a wall presented significantly lower scores than the same test without the wall (*p* < 0.001). ICC analyses indicate, on average, moderate correlations between the scores of the classic BBT and the vBBT, for both the 6 zones versions without a wall (ICC = 0.31) and with a wall (ICC = 0.48) (Table 3).

### 3.3. Study Phase 2

In phase 2, we observed a significant difference between the BBT and the three versions of the vBBT (*p* < 0.001) (Table 3, Figure 2). Moreover, the different versions of the BBT generated scores that were all significantly different from one another (*p* < 0.001). In order of increasing difficulty (from the easiest test generating the highest scores to the most difficult test generating the lowest scores), we find the following BBT versions: vBBT-free zone, vBBT-4 zones, original BBT, and vBBT-6 zones.

**Figure 3:**
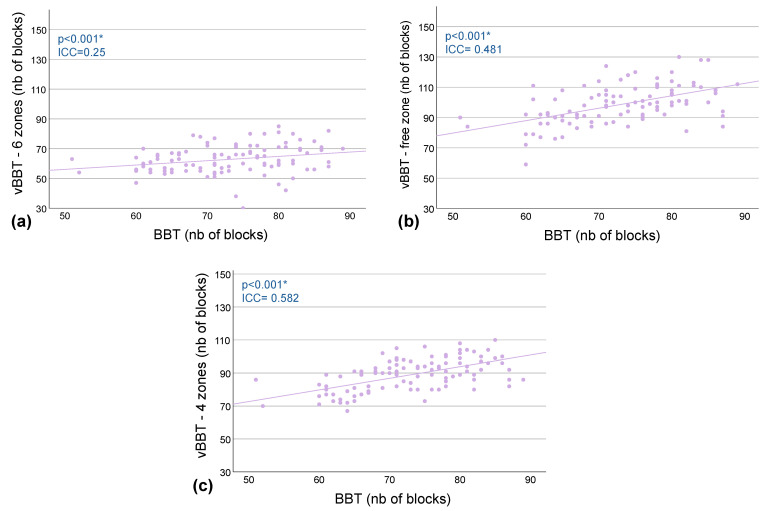
Correlations between BBT and vBBTs for healthy participants. Each dot represents a participant’s score, with the original BBT on the x-axis and a vBBT version on the y-axis, illustrating the relationship between the two assessments. The fitted line highlights the overall trend of the relationship. Scatter plots display participants’ scores on the BBT and (**a**) the vBBT-6 zones, (**b**) the vBBT-free zone, and (**c**) the vBBT-4 zones. *vBBT = virtual Box and Block Test, nb = number,* * = *p* < 0.001.

Most of the ICC analyses indicate small to medium correlations between the scores of the vBBT and the classic BBT (ICC< 0.48) (Table 3). However, a notable exception is observed in the 4 zones configuration of the vBBT, where a large correlation is demonstrated, with an ICC value of 0.58 (Table 3, Figure 3).

A significant but small correlation was found between the scores obtained in the ToL and those obtained with the vBBT-6 zones (*p* = 0.007, r = −0.284) (Table 3). This relationship indicates that individuals who demonstrated more efficient planning abilities (shorter planning time) tended to achieve higher scores on the vBBT-6 zones. On the other hand, the other virtual versions and the BBT showed no correlation with the ToL (*p* ≥ 0.126) (Table 3).

The error rates on the vBBT-4 zones and the vBBT-free zone were 1.32% and 7.84%, respectively.

### 3.4. Study Phase 3

**Figure 4:**
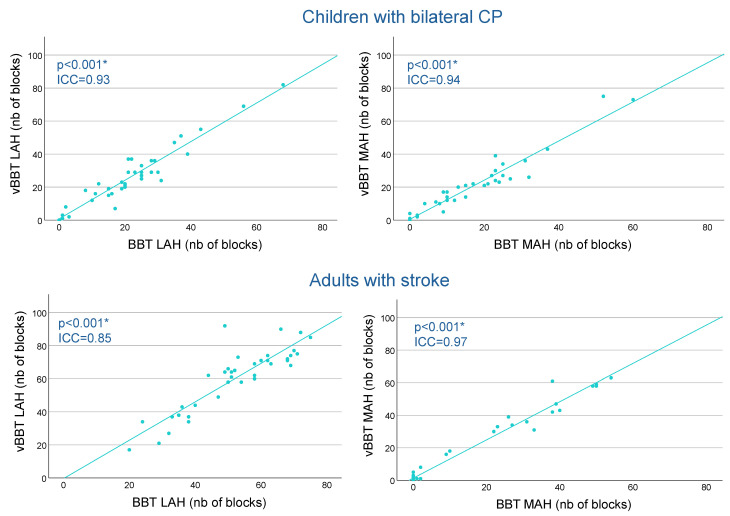
**Correlations between BBT and vBBT-4 zones for children with bilateral CP and adults with stroke.** Each dot represents a participant’s score, with the original Box and Block Test (BBT) on the x-axis and vBBT-4 zones on the y-axis, illustrating the relationship between the two assessments. The fitted line highlights the overall trend of the relationship. Scatter plots display participants’ scores on the BBT and the vBBT-4 zones for the LAH (**left** panels) and the MAH (**right** panels), in children with bilateral cerebral palsy (CP) (**upper** panels) and adults with stroke (**lower** panels). * = *p* < 0.001.

The ICC analyses indicate large correlations between the scores of the vBBT-4 zones and the BBT for both MAH and LAH for children with CP and adults with stroke (ICC ≥ 0.85) (Table 3, Figure 4)

For children with bilateral CP, we observed large correlations between the scores obtained in the ABILHAND-Kids and the vBBT-4 zones for both the MAH and the LAH (both *p* < 0.001, r ≥ 0.71). Similar correlations were found between the original BBT and ABILHAND-Kids questionnaire for both hands (*p* < 0.001, r ≥ 0.72). For adults with stroke, we observed a large correlation for the MAH (*p* < 0.001, r = 0.79) but not for the LAH (*p* = 0.07, r = 0.31) between the scores obtained in the ABILHAND-CS and the vBBT-4 zones. Similar correlations were found between the original BBT and ABILHAND questionnaire for MAH (*p* < 0.001, r = 0.77) and LAH (*p* = 0.12, r = 0.27).

## 4. Discussion

The primary objective of this study was to develop and validate a virtual version of the BBT to enable remote assessment of gross manual dexterity. The first phase of the study focused on creating two versions of the vBBT, both incorporating six distinct zones, but differing in the height of the separation between the areas. While the high-wall version demonstrated a moderate intraclass correlation with the BBT, it posed ergonomic challenges, requiring excessive shoulder internal rotation combined with shoulder flexion, which was both uncomfortable and seemed to have an influence on the obtained scores (notably, by reducing them). This difference might be due to the constraint of placing and picking precisely the block on the screen rather than only releasing the block after passing the central divider in the original BBT. A previous study already compared this vBBT-6 zones version using the separating wall with the BBT [29]. In this study, clinicians rated the difficulty of this version relative to the traditional BBT in terms of motor demands, with the only aspect deemed more difficult in the high-wall version being “arm movement required to displace the cube”, reinforcing our observations. Based on those results, using a simple bar to separate both areas seems more appropriate. However, its moderate intraclass correlation with the BBT raised the hypothesis of a potential influence of other confounding variables, such as a spatial planning component, which are absent in the initial BBT. Spatial planning is a fundamental component of human cognition, encompassing the ability to organize and execute actions within space. Placing an object with precision and speed requires planning and the engagement of additional cognitive resources [30]. Participants adopted various strategies during the vBBT-6 zones task: some consistently placed blocks in the same zones, others randomly placed them, some initiated without a discernible strategy but developed one throughout the test, others prioritized blocks on the periphery before transitioning to the center, etc. These differences in strategies may influence test scores independently of manual dexterity, highlighting the potential role of spatial planning in the task [24,31].

To explore this executive function, the ToL task was included in the second phase of the study, alongside the development of two additional vBBT versions with fewer zones: a 4-zones version and a version with no predefined zones. Interestingly, the significant intraclass correlation observed in this phase between the ToL score and the vBBT-6 zones suggests a possible influence of planning ability on vBBT-6 zones performance, although this association was weak and should be interpreted with caution. In contrast, no intraclass correlation was found between the ToL scores and the scores of the 4 zones or free zone vBBT versions, recommending the use of those simplified versions. Moreover, the vBBT-4 zones version exhibited the largest intraclass correlation with the original BBT (ICC = 0.58), along with the lowest blocks displacement detection error rate (1.4%), suggesting this version to be the most suitable virtual version based on the original BBT.

In the third validation phase, when tested in children with CP and post-stroke adults, the vBBT-4 zones demonstrated excellent intraclass correlations with the original BBT (ICC ≥ 0.93 for children and ICC ≥ 0.85 for adults). Additionally, these results are further supported by the concomitant significant correlations observed between the manual dexterity scores obtained on the vBBT/BBT and the measure derived from the ABILHAND questionnaires. These results align with the existing literature highlighting a meaningful correlation between ABILHAND and BBT (r ≥ 0.481, *p* < 0.001) [27,32,33,34], further supporting the potential of the vBBT-4 zones as a valid tool for remote assessment of unilateral gross manual dexterity.

Despite the conclusive results leading to the recommendation for using the vBBT-4 zones version, the obtained scores significantly differ from the BBT in favor of the virtual test. These differences in scores highlight the fact that the vBBT-4 zones cannot be used interchangeably with the original BBT for clinical interpretation or longitudinal follow-up. Although both tests aim to assess gross manual dexterity, their structural and motor demands differ. Therefore, clinicians should treat them as distinct tools, each requiring specific normative references. This observed divergence might be explained by differences in the grasping and lifting/transporting phases of tasks between the two versions. Regarding the grasping phase, the vBBT lacks the “digging phase” inherent to the BBT, where participants are required to locate and grasp a single block among the 150 available in the box [22]. In contrast, the vBBT presents only four blocks on the screen, which can be directly accessed without moving other blocks. This “digging phase” is likely to increase the motor demand of the BBT, contributing to lower scores in comparison with the vBBT. Additionally, the lifting/transporting phase also differs slightly between the two versions. In the vBBT, the lower height of the bar might reduce the needs of shoulder flexion/external rotation but might also increase the needs of shoulder adduction or elbow extension, both potentially influencing the observed scores on both tests. Even though these discrepancies in task performance should be more precisely investigated, the observed difference between the scores highlights the need to establish normative values tailored to this new version of the vBBT.

### 4.1. Clinical Interest in vBBT

In the literature, numerous alternative versions of the BBT have been developed, employing a wide range of technologies. Some versions use immersive virtual reality with hand-held controllers or haptic devices [29,35,36], while others rely more on hand-tracking systems with real object manipulation [29,37]. Additionally, non-immersive versions based on tablets or standard computers have been introduced [29,38,39,40,41].

In contrast to some of the previously developed vBBTs, the design of this newly developed vBBT-4 zones on the REAtouch^®^ takes place in a broader context of the implementation of a home-based telerehabilitation program already including the use of the device [18,19]. This newly developed version might then be used to assess gross unimanual dexterity at home for patients involved in such a telerehabilitation program, or in rehabilitation centers that invested in this virtual device designed for rehabilitation when a traditional BBT is not available. User satisfaction, accessibility, suitability for home-based use, and long-term feasibility of the REAtouch^®^ device are currently being investigated in a separate randomized controlled trial focusing on the broader implementation of the system in home-based telerehabilitation.

In terms of validity, the vBBT developed in the current study aligns broadly with the literature, as evidenced by large correlations (r > 0.5) also reported between physical and virtual scores in previous studies [29,38,40]. These findings support the validity of virtual versions of the BBT for assessing manual dexterity across both healthy and clinical populations (e.g., post-stroke or other neurological disorders). However, studies consistently report lower scores in virtual versions compared to the BBT, contrasting with our results [29,36,37,38,39,40]. This discrepancy may be explained by two main factors absent from the vBBT developed in the current study: the complexity introduced by the immersive nature of virtual versions and the lack of sensory feedback due to the absence of physical object manipulation.

First, the immersive nature of certain virtual versions may present greater challenges for some individuals. This could be attributed to the altered perception of depth in virtual environments, which differs significantly from real-world experiences. For populations affected by conditions such as apraxia, vertigo, epilepsy, or other cognitive impairments, immersive virtual reality can pose substantial difficulties [42]. In our study, only the 6 zones condition, with or without the wall, produced lower scores than the BBT, behaving similarly to virtual versions described in the literature. Interestingly, a small but significant correlation was observed between this version and performance on the ToL task (*p* = 0.007, r = –0.28), suggesting that planning ability may play a minor role in vBBT-6 zones’ performance. This raises the possibility that, like some other virtual BBTs in the literature, performance on such tasks could be partly influenced by additional cognitive dimensions such as planning or visuospatial processing. However, given the modest strength of the correlation, this interpretation should be considered with caution.

Second, the challenge of maintaining sensory feedback with real-life objects during virtual reality use remains a critical issue. In the context of the BBT, the physical properties of the blocks—such as their shape, weight, and texture—can influence grasping strategies, thereby impacting the assessment scores [43,44]. Absence of such haptic/sensory feedback observed in much of the virtual versions of the BBT has been described as a major limitation, not only diminishing user experience but also increasing task difficulty for individuals with motor or cognitive impairments [29,39,40]. The critical role of multisensory feedback is further highlighted by enhanced reaction times and task performance in virtual environments incorporating haptic feedback [45]. In addition, participants using a non-immersive virtual version of the BBT without tangible objects to manipulate reported difficulties in grasping virtual blocks with preferences for a physical BBT [39]. To address the absence of tactile feedback, Dong et al. developed a haptic feedback device (Omega.7) capable of delivering realistic force sensations [36]. However, such systems might still present difficulties replicating the authentic tactile experience of manipulating physical blocks, with additional questioning about the real added value and clinical usefulness of over-equipped virtual versions of a test compared to its initial procedure. On the other hand, some of the virtual BBT versions proposed in the literature suggest the use of tangible objects with hand-held controllers (e.g., handles). Even these allow real sensory feedback, and the physical characteristics of controllers (weight and size) and the way to interact with the environment (pressing buttons) might exacerbate challenges for individuals with upper limb impairments. Additionally, the potential complexity of understanding controller functionality and the gap between the movement needed to grasp a real block or press a button may pose additional barriers [29]. By maintaining the use of real blocks manipulation in a non-immersive environment, the vBBT-4 zones developed in this study bypass these potential limitations, preserving a concrete sensory experience that seems critical for valid, accurate, and meaningful assessments.

In addition, the vBBT-4 zones demonstrated possible use for a wide range of motor profiles. In our sample, mRS scores ranged from 1 to 5 in stroke participants, and GMFCS and MACS levels ranged from I to V and I to IV, respectively, in children with CP. Despite these varying degrees of physical impairment, all participants were able to complete the test, further supporting its clinical utility. The vBBT-4 zones also appeared accessible to individuals with cognitive impairments. Stroke participants in our study had MoCA scores ranging from 12 to 30, yet all were able to understand and complete the task. This reinforces the idea that the test is suitable for populations with reduced cognitive capacities, particularly when administered with brief, clear standardized instructions from the examiner. Finally, although we did not conduct a formal learning effect analysis, the vBBT-4 zones included a short, standardized tutorial (with both written and audio guidance) and a practice trial with 16 blocks. All participants completed the test on their first attempt without the need for repeated explanations. These elements suggest that the vBBT-4 zones version has a minimal learning curve and can be easily administered across a wide range of users, including in supervised home-based contexts.

### 4.2. Limitations

The present study has some limitations that may have impacted the results. The participants’ prior experience with virtual environments or digital interfaces was not assessed, and no specific measure of user experience or ergonomics was included, which may have introduced variability in task performance unrelated to motor skills per se. Furthermore, no formal evaluation of user comfort during task execution was conducted. In addition, the spatial configuration of the virtual interface, particularly the lateral positioning of some of the zones, may have unintentionally induced different biomechanical demands, such as shoulder adduction and elbow extension. These factors may have influenced motor strategies in ways that differ from the original BBT.

## 5. Conclusions

In conclusion, the vBBT-4 zones version, with a combination of a virtual environment, real object manipulation, and a design very close to the original BBT, represents a valid and practical alternative to the BBT for remote dexterity assessment. While further research is needed to establish normative data, the strong performance of the vBBT-4 zones across both healthy and clinical populations underscores its potential usefulness in various settings.

Future studies should aim to establish new normative datasets, controlling for key demographic variables such as age and gender, and evaluate the test–retest reliability of the vBBT, as only concurrent validity was assessed in the present study. Moreover, integrating standardized assessments of digital familiarity (e.g., the Media and Technology Usage and Attitudes Scale), user experience (e.g., self-report questionnaires or interviews), and ergonomics or usability (e.g., the System Usability Scale) would help disentangle motor performance from contextual or perceptual influences.

Additional perspectives might also explore the adaptability of the software by using various objects to assess different aspects of dexterity. This flexibility could enable the evaluation of a broader spectrum of hand functions, offering valuable insights into different types of prehension. Comparing performance across these test variants could further refine our understanding of their similarities and differences in assessing upper limb motor skills.

## Figures and Tables

**Table 2:** Demographic data.

	*n*	Age Mean (Range)	Gender (F/M)	Laterality/LAH (R/L)
**Phase 1. Hardware definition (with or without separating wall)**				
Healthy participants	100	23.25 (18–60)	67/33	94/6
**Phase 2. Software definition (6 zones, 4 zones, free zone)**				
Healthy participants	105	21.3 (18–43)	63/42	82/23
**Phase 3. Validation in clinical populations (concurrent validity)**				
Children with bilateral CP	37	9.6 (5–18)	13/24	20/17
Adults with stroke	37	61.7 (31–80)	17/20	22/15

*F = Female; M = Male; LAH = Less Affected Hand; R = Right; L = Left; CP = Cerebral Palsy.*

**Table 3:** Results.

		Median [Q1–Q3]	Friedman’s ANOVA		ICC with BBT		Correlations	
			*p*-Value	Holm–Sidak Post-Hoc (Difference with BBT)	*p*-Value	Intraclass Correlation [95% CI]	*p*-Value	r
**Phase 1. Hardware definition (with or without separating wall) (*n* = 100)**								
	BBT	70.5 [66–75]	<0.001 **	*-*	-	-	-	-
	vBBT-6 zones	67 [63–71]		<0.001 **	<0.001 **	0.31 [0.13; 0.48]	-	-
	vBBT-6 zones with wall	51 [48–56]		<0.001 **	<0.001 **	0.48 [0.31; 0.62]	-	-
**Phase 2. Software definition (6 zones, 4 zones, free zone) (*n* = 105)**								
							**with Tower of London** **(planning time) ^1^**	
	BBT	74 [66.5–80]	<0.001 **	-	-	-	0.272	−0.12
	vBBT-6 zones	62 [56–69]		<0.001 **	<0.001 **	0.25 [0.06; 0.42]	0.007 *	−0.28
	vBBT-4 zones	90 [81–96]		<0.001 **	<0.001 **	0.58 [0.44; 0.70]	0.126	−0.16
	vBBT-free zone	99 [90.5–108]		<0.001 **	<0.001 **	0.48 [0.32; 0.62]	0.317	−0.11
**Phase 3. Validation in clinical populations (concurrent validity): children with bilateral CP (*n* = 37) and adults with stroke (*n* = 37)**								
							**with ABILHAND** **questionnaires**	
BCP	BBT LAH	21 [13.5–28.5]	-	-	-	-	<0.001 **	0.72 ^1^
	BBT MAH	15 [5.5–24]	-	-	-	-	<0.001 **	0.81 ^1^
	vBBT-4 zones LAH	24 [16–36]	-	-	<0.001 **	0.93 [0.87; 0.96]	<0.001 **	0.71 ^1^
	vBBT-4 zones MAH	20 [7.5–26.5]	-	-	<0.001 **	0.94 [0.89; 0.97]	<0.001 **	0.76 ^1^
Stroke	BBT LAH	52 [39–64.5]	-	-	-	-	0.115	0.27 ^2^
	BBT MAH	1 [0–32]	-	-	-	-	<0.001 **	0.77 ^1^
	vBBT-4 zones LAH	64 [43.5–72.5]	-	-	<0.001 **	0.85 [0.73; 0.92]	0.069	0.31 ^2^
	vBBT-4 zones MAH	3 [0–37.5]	-	-	<0.001 **	0.97 [0.94; 0.98]	<0.001 **	0.79 ^1^

ICC: Intraclass Correlation Coefficient, BBT = Box and Block Test, vBBT = virutal Box and Block Test, LAH = Less-Affected Hand, MAH = More-Affected Hand, CI = Confidence Interval, ** p < 0.001, * p < 0.05, Spearman Correlation^1^, *Pearson Correlation*^2^.

## Data Availability

Data supporting reported results can be found in Supplementary Material Files S1–S4.

## Supplementary Materials

The Supplementary Materials contain the raw scores for the original BBT, vBBT-6 zones, vBBT-6 zones with wall, ToL (planning), ABILHAND (expressed as % of logits), age, and laterality or most affected hand. The following supporting information can be downloaded at: https://www.mdpi.com/article/10.3390/bioengineering12060662/s1, File S1: Results phase 1, File S2: Results phase 2, File S3: Results phase 3—BCP, and File S4: Results phase 3—Stroke.

## Abbreviations

The following abbreviations are used in this manuscript:

BBT	Box and Block Test
CIMT	Constraint-Induced Movement Therapy
CP	Cerebral Palsy
GMFCS	Gross Motor Function Classification System
HABIT	Hand–Arm Bimanual Intensive Therapy
HABIT-ILE	Hand–Arm Bimanual Intensive Therapy Including Lower Extremities
ICC	Intraclass Correlation Coefficient
LAH	Less-Affected Hand
MACS	Manual Ability Classification System
MAH	More-Affected Hand
MoCA	Montreal Cognitive Assessment
mRS	Modified Rankin Scale
ToL	Tower of London
vBBT	Virtual Box and Block Test
