# Supplementary material for: Development and Validation of a Virtual Version of the Box and Block Test to Assess Manual Dexterity at Home for Adults with Stroke and Children with Cerebral Palsy
Source: Bioengineering (Basel). 2025 Jun 16;12(6):662. doi: 10.3390/bioengineering12060662 (PMC12189916; doi:10.3390/bioengineering12060662)
Supplement: Supplementary file 1 [file bioengineering-12-00662-s001.zip › Supplementary material File S1 (results phase 1).pdf]

	original BBT	vBBT-6 zones	vBBT-6 zones with wall
1	70	85	56
2	66	85	59
3	70	60	46
4	84	71	52
5	75	71	56
6	80	72	54
7	67	58	48
8	77	53	42
9	60	58	45
10	53	75	50
11	74	71	50
12	83	74	56
13	78	71	43
14	73	72	62
15	60	60	48
16	74	65	51
17	66	67	56
18	67	67	48
19	71	59	48
20	80	73	57
21	82	70	60
22	78	66	51
23	69	67	51
24	54	68	48
25	66	65	49
26	73	66	51
27	74	64	53
28	82	70	56
29	71	61	54
30	62	50	49
31	62	67	48
32	63	67	53
33	67	62	51
34	75	60	48
35	78	79	58
36	71	68	52
37	56	67	44
38	73	79	65
39	78	71	50
40	74	72	61
41	68	67	50
42	64	65	47
43	66	53	45
44	59	69	51
45	71	66	54

46	66	57	45
47	55	67	49
48	66	72	54
49	67	69	51
50	76	80	61
51	87	75	63
52	91	71	58
53	75	76	51
54	69	79	55
55	73	69	53
56	65	63	46
57	80	66	53
58	82	75	62
59	75	68	55
60	58	57	42
61	69	62	45
62	74	71	52
63	80	66	53
64	75	76	51
65	69	66	48
66	62	71	49
67	76	78	61
68	90	80	58
69	76	67	51
70	80	70	53
71	68	75	57
72	69	76	61
73	66	61	51
74	68	77	57
75	69	71	57
76	75	70	52
77	69	61	45
78	83	69	60
79	72	64	56
80	70	60	53
81	73	60	44
82	72	70	47
83	71	65	49
84	86	67	60
85	59	59	43
86	72	58	48
87	62	63	52
88	73	75	58
89	61	64	41
90	58	71	47
91	73	65	44

92	74	66	47
93	70	65	56
94	65	63	54
95	70	68	52
96	61	66	44
97	69	62	49
98	70	61	55
99	69	61	50
100	67	68	52
