# Supplementary material for: Development and Validation of a Virtual Version of the Box and Block Test to Assess Manual Dexterity at Home for Adults with Stroke and Children with Cerebral Palsy
Source: Bioengineering (Basel). 2025 Jun 16;12(6):662. doi: 10.3390/bioengineering12060662 (PMC12189916; doi:10.3390/bioengineering12060662)
Supplement: Supplementary file 1 [file bioengineering-12-00662-s001.zip › Supplementary material File S2 (results phase 2).pdf]

Participants	Gender	Laterality	Age	original BBT	vBBT 6 zones	vBBT free zone	vBBT 4 zones	ToL (planning)
1	M	R	43	66	66	86	91	2.5
2	M	R	18	65	67	88	91	5.64
3	F	R	25	85	75	128	110	2.91
4	F	R	18	74	71	84	80	4.38
5	F	R	19	61	60	79	80	11.95
6	F	R	20	70	72	91	90	3.31
7	F	R	19	74	38	118	93	5.13
8	F	R	22	60	55	59	76	15.34
9	M	R	23	52	54	84	70	
10	F	L	23	75	66	120	106	5.21
11	M	R	23	51	63	90	86	
12	M	R	24	78	80	116	100	
13	M	L	23	80	59	105	108	
14	F	R	19	72	57	87	85	4.47
15	M	L	19	74	72	98	93	
16	F	R	20	71	64	108	98	
17	M	R	21	69	78	87	102	3.07
18	F	R	23	82	80	99	103	2.5
19	F	R	21	69	57	103	90	10.36
20	F	L	23	76	56	91	96	10.36
21	F	R	19	81	42	101	91	3.09
22	M	R	19	73	64	115	84	
23	F	R	20	73	58	92	97	1.64
24	F	R	19	83	69	113	100	
25	F	L	18	70	51	105	97	
26	M	R	23	69	55	84	81	5.03
27	F	R	23	68	59	91	91	2.4
28	F	R	21	65	62	108	81	4.87
29	F	L	24	64	53	76	67	7.9
30	F	R	21	75	30	109	94	4.25
31	F	R	20	71	77	108	99	3.43
32	F	R	20	71	60	97	89	
33	F	R	22	76	67	89	92	
34	F	R	20	86	69	108	96	
35	M	R	22	73	55	104	80	1.65
36	M	L	21	71	54	101	91	
37	M	R	20	80	71	100	99	
38	F	R	22	83	70	111	92	4.02
39	M	R	18	77	65	99	94	1.59
40	M	R	22	61	58	102	77	7.29
41	F	R	32	85	56	100	99	2.85
42	F	R	20	65	54	77	73	3.29
43	M	R	24	72	66	100	93	2.49
44	F	L	19	80	85	114	104	
45	F	R	22	79	58	92	88	4.04
46	F	L	18	76	67	101	80	7.46
47	M	L	19	80	60	108	102	

48	F	R	19	71	53	105	82	5.93
49	M	R	22	72	54	104	98	2.23
50	M	R	21	81	71	111	94	2.04
51	F	R	21	65	56	91	76	6.1
52	F	L	19	62	54	92	73	5.73
53	F	R	41	74	66	94	88	6.05
54	F	R	18	64	55	90	72	4.38
55	F	R	18	68	59	98	90	5.78
56	M	R	18	66	67	94	89	5.83
57	F	R	18	83	76	111	97	1.49
58	M	R	18	67	59	92	78	
59	M	R	21	87	58	84	92	11.97
60	M	R	19	71	59	124	105	6.23
61	F	R	21	78	60	111	96	4.21
62	F	R	19	77	64	104	80	5.39
63	F	R	19	71	66	98	95	6.73
64	M	R	22	84	56	128	96	5.07
65	M	R	22	61	59	79	82	11.03
66	F	L	31	78	52	112	91	6.61
67	F	R	18	67	68	90	82	2.26
68	F	R	18	76	68	97	89	1.8
69	F	R	21	60	56	79	83	2.4
70	F	R	21	67	55	83	79	2.34
71	M	R	19	78	60	97	101	5.23
72	M	R	22	74	73	94	94	3.28
73	F	R	30	78	69	100	87	3.87
74	F	R	34	80	62	105	96	2.53
75	F	R	19	60	47	72	71	19.52
76	M	R	19	64	57	102	79	5.04
77	M	R	23	87	61	91	86	8.26
78	F	R	22	82	50	101	86	6.71
79	M	L	20	76	80	101	92	5.63
80	M	R	19	70	74	114	93	3.64
81	M	R	21	71	51	86	82	2.58
82	F	R	20	78	65	110	85	5.04
83	F	R	19	86	71	106	100	10.89
84	F	R	18	62	61	86	77	1.94
85	F	R	19	63	65	86	88	6.56
86	F	L	19	63	63	93	74	5.47
87	M	R	19	66	63	86	77	5.85
88	M	R	19	63	66	92	72	3.89
89	F	R	19	82	60	81	80	3.96
90	F	L	18	62	56	77	77	2.23
91	F	R	20	60	64	92	71	1.67
92	F	L	19	75	60	100	73	1.82
93	F	L	21	78	59	95	82	1.94
94	F	R	21	80	81	106	102	5.05
95	M	R	19	87	82	94	82	1.64
96	M	R	20	81	74	130	104	2.89
97	M	R	20	76	72	92	100	1.17

98	M	L	25	77	72	104	93	2.1
99	F	L	23	80	46	98	99	4.19
100	M	R	21	80	62	120	89	5.55
101	F	R	23	82	62	101	89	7.83
102	F	L	21	61	70	111	89	1.76
103	M	L	22	68	79	111	93	2.53
104	M	L	23	84	67	110	104	2.67
105	M	L	22	89	70	112	86	3.05
