# Supplementary material for: Development and Validation of a Virtual Version of the Box and Block Test to Assess Manual Dexterity at Home for Adults with Stroke and Children with Cerebral Palsy
Source: Bioengineering (Basel). 2025 Jun 16;12(6):662. doi: 10.3390/bioengineering12060662 (PMC12189916; doi:10.3390/bioengineering12060662)
Supplement: Supplementary file 1 [file bioengineering-12-00662-s001.zip › Supplementary material File S3 (results phase 3) BCP.pdf]

participants ID	original BBT LAH	original BBT MAH	vBBT LAH	vBBT MAH	ABILHAND (% of logit)	Age	MAH
02.00.01.23	37	27	51	25	61.5	5	L
02.00.02.23	68	60	82	73	61.5	6	L
02.00.03.23	21	2	29	2	34.1	13	L
02.00.04.23	2	0	8	1	28.7	10	L
02.01.01.23	3	0	2	0	0	15	L
02.01.02.23	15	15	19	21	38.6	8	L
02.01.03.23	39	31	40	36	68	5	L
02.01.04.23	21	23	37	30	55.2	7	R
02.02.01.23	1	0	1	0	21.3	10	R
02.02.02.23	17	9	7	5	42	14	R
02.02.03.23	29	25	36	34	52.7	11	R
02.02.04.23	31	32	24	26	58.3	10	R
02.03.01.23	8	8	18	10	38.6	5	R
02.03.02.23	25	17	27	22	37.1	7	R
02.03.03.23	30	24	29	23	49	10	R
02.03.04.23	28	21	36	22	41.6	18	R
02.03.05.23	16	9	16	17	28.9	8	L
02.03.06.23	0	0	0	0	29	5	L
02.00.01.24	28	24	29	23	63.2	7	R
02.00.03.24	25	23	25	24	48.5	9	L
02.00.05.24	20	7	21	11	26.6	10	L
02.01.01.24	25	4	29	10	51.5	12	R
02.01.02.24	19	0	23	4	32.4	11	L
02.01.03.24	22	23	37	39	44.2	6	L
02.01.04.24	56	52	69	75	59.1	14	L
02.01.05.24	1	2	3	3	28.7	7	R
02.01.06.24	15	0	15	1	40	11	L
02.02.01.24	10	15	12	14	49.9	8	L
02.02.02.24	20	10	22	17	46.2	16	L
02.02.05.24	11	12	16	12	46.5	9	R
02.02.06.24	23	22	29	27	65.5	9	R
02.03.01.24	20	20	20	21	54.8	8	L
02.03.02.24	19	10	19	14	42.7	6	L
02.03.03.24	25	25	33	27	42.7	9	R
02.03.04.24	35	37	47	43	71.7	18	L
02.03.05.24	12	10	22	12	58	9	R
02.03.06.24	43	13	55	20	54	9	R
