# Supplementary material for: Development and Validation of a Virtual Version of the Box and Block Test to Assess Manual Dexterity at Home for Adults with Stroke and Children with Cerebral Palsy
Source: Bioengineering (Basel). 2025 Jun 16;12(6):662. doi: 10.3390/bioengineering12060662 (PMC12189916; doi:10.3390/bioengineering12060662)
Supplement: Supplementary file 1 [file bioengineering-12-00662-s001.zip › Supplementary material File S4 (results phase 3) stroke.pdf]

participants ID	original BBT LAH	original BBT MAH	vBBT LAH	vBBT MAH	ABILHAND (% of logit)	Age	MAH	Gender
000123	71	0	75	3	56.5	65	R	M
000223	38	0	34	0	30.7	56	R	F
000323	60	0	71	0	39.3	42	L	F
000423	68	50	72	59	60.8	62	L	F
010123	69	50	74	58	100	43	L	M
010223	32	33	27	31	53.1	76	L	F
010323	44	9	62	16	52.4	66	R	F
010423	70	31	77	36	59.9	46	L	F
020123	50	0	66	5	37.7	69	L	M
020223	66	0	90	2	58.7	59	L	M
020323	63	1	69	1	55.6	51	L	M
020423	53	38	73	61	62.7	86	L	M
030123	58	40	62	43	84.1	60	L	M
030223	62	22	71	30	45.7	80	R	F
030323	51	49	61	58	73.7	63	L	M
030423	49	27	92	34	56.5	54	R	M
030523	47	0	49	0	56.3	31	L	M
000124	29	0	21	0		55	L	F
000224	20	0	17	0	18.5	60	L	F
000324	58	0	69	0	39.8	80	R	F
000424	50	0	58	0	45.7	71	L	M
000624	40	0	44	0	46.6	72	R	M
010124	38	2	37	8	58.1	47	R	F
010224	58	10	60	18	46.1	64	L	M
010324	69	0	68	0	36.3	53	L	M
010424	51	0	64	0	41.6	78	R	M
010524	68	0	71	1	41.6	72	R	F
020124	36	0	43	0	43.9	71	R	M
020224	62	2	74	1		80	L	F
020324	35	0	38	0	30.1	74	L	F
020524	52	39	65	47	53.5	42	R	M
030124	72	23	88	33	56.7	51	R	M
030224	49	54	64	63	77.1	66	L	M
030324	24	26	34	39	75.8	73	R	M
030424	33	0	37	0	47.7	61	R	F
030524	54	38	58	42	60.9	57	L	F
030624	75	0	85	0	54.5	48	L	F
